# Editorial: Insights in glaucoma: 2023

**DOI:** 10.3389/fopht.2024.1519088

**Published:** 2025-01-08

**Authors:** Claire L. Peterson, Tina T. Wong, Shamira Perera

**Affiliations:** Singapore National Eye Centre, Singapore Eye Research Institute, Singapore, Eye-ACP, Duke-NUS Medical School, Singapore, Singapore

**Keywords:** cataract surgery, cataract surgery and glaucoma, minimally invasive glaucoma surgery (MIGS), EDOF IOL, extended depth of focus (EDOF), premium intraocular lenses

*Extended Depth of Focus Intraocular Lenses – the answer to achieving spectacle-independence for Glaucoma patients?*

Cataract and glaucoma are two of the most common eye conditions that lead to vision impairment in older adults and often coexist in this increasing group of patients. The constant evolution of intraocular lens (IOL) technology has enabled us to offer our patients a wider range of refractive options that promise spectacle-independence. However, IOLs that use diffractive technology to establish multiple foci are associated with glare, haloes, reduction in contrast sensitivity and a decrease in visual field sensitivity (e.g. TECNIS Multifocal ZLBOO, Johnson & Johnson Vision, Santa Ana, CA, USA; AcrySof IQ PanoOptix, Alcon, Fort Worth, TX, USA) (1, 2). Glaucoma patients, who often have reduced contrast sensitivity and night vision would be poor candidates for multifocal or trifocal IOLs, due to concerns over further decreasing their visual quality (3). Additionally, the treatment of glaucoma itself renders such patients unsuitable for multifocal options. First, many patients are on ocular hypotensive drops, and concurrently have a higher incidence of ocular surface disorders which may further hinder contrast sensitivity (4, 5). Second, glaucoma filtering surgeries may result in astigmatism post-operatively which can lead to a greater deterioration of visual acuity at all distances for multifocal compared to monofocal IOLs (6, 7). Third, secondary glaucomas such as PXF often present with an associated capsular bag instability, which may render multifocal options less appropriate due to the greater possibility of IOL displacement.

## Types of EDOF IOLs

New extended depth of focus (EDOF) IOL are gaining popularity since their introduction in 2014, as they offer improved intermediate vision and reduced reliance of glasses, have significantly less optical phenomena, and less impact on contrast sensitivity and visual field sensitivity compared to diffractive multifocal IOLs (1). EDOF technologies include small-aperture (e.g. IC-8, AcuFocus, Inc, Irvine, CA, USA), bioanalogic hydrogel (eg. Wichterle IOL-Continuous Focus, Medicem, Kamenne Zehrovice, Czech Republic), diffractive (e.g. Tecnis Symfony, Johnson & Johnson Vision, Santa Ana, CA, USA; AT LARA 829MP, Carl Zeiss Meditec AG, Jena, Germany) and newer non-diffractive wavefront-shaping (eg. Acrysof IQ Vivity, Alcon, Fort Worth, TX, USA) designs (8, 9). A study by Asena et al. comparing visual performance and quality of life outcomes following bilateral non-diffractive EDOF and trifocal IOLs found that while EDOF IOLs performed better for distance and trifocal IOLs better for near, the overall reported quality of life (Visual Function Index-14) and quality of vision (QoV) questionnaire results were better for the EDOF IOL (10). Another study of non-diffractive EDOF IOLs by Kohnen et al. found that the most common optical phenomenon were glare and haloes (25%) followed by ghosting (7%), with good QoV outcomes for day and night driving, watching TV, cooking, computer and domestic work (11). A newer purely refractive EDOF IOL has also been launched (TECNIS PureSEE, Johnson and Johnson Vision, Santa Ana, CA, USA), which is designed to provide a smooth increased range of vision but with only infrequent comparable visual side effects to monofocal IOLs (12). A study comparing bilateral implantation of the TECNIS PureSee EDOF IOL with the TECNIS Eyhance monofocal IOL (Johnson and Johnson Vision, Santa Ana, CA, USA), found that the EDOF IOL had extended range of vision with similar distance visual acuity, contrast sensitivity and dysphotopsia compared to the monofocal IOL (13).

## Mini-monovision using EDOF IOLs

Mini-monovision using EDOF IOLs is also an option for patients undergoing bilateral cataract surgery. In a study by van Amelsfort et al., patients received bilateral non-diffractive EDOF IOL (AcrySof IQ Vivity, Alcon, Fort Worth, TX, USA), and were targeted for mini-monovision, with the dominant eye aimed for emmetropia and the non-dominant eye aimed between -0.25D and -0.50D. The reported percentage of patients not or rarely using spectacles for distance, intermediate and near distances were 96%, 68% and 38% respectively, with >90% of patients reporting no haloes, glares or starbursts, and a high satisfaction of daily life activities on the Dutch Catquest-9SF score (14). Another study by Tomagova et al. of patients undergoing bilateral non-diffractive EDOF IOL implantation (BVI Isopure, PhysIOL, Liege Belgium) with mini-monovision targeting -0.50D, undergoing bilateral cataract surgery found that this resulted in spectacle-independence for intermediate vision in 95% of their cohort and 34% of their cohort for near vision, which is higher compared to other studies without mini-monovision (15, 16). On a practical note, it is important that to achieve the refractive target for both eyes (dominant eye emmetropia, non-dominant eye -0.25 to -0.50D) for mini-monovision to work optimally. This is because a myopic shift of the non-dominant eye will create monovision with more anisometropia, which has been show to decrease stereopsis, contrast sensitivity and patient satisfaction (17, 18).

## EDOF IOLs for glaucoma patients

There are limited studies in literature on EDOF IOL implantation in glaucoma patients; most focus on patients with mild primary open angle glaucoma (POAG) and report positive outcomes. A study by Kerr et al. compared bilaterally implanted EDOF IOL (Acrysof IQ Vivity, Alcon, Fort Worth, TX, USA) with monofocal IOL (Clareon/SN6AT/SN60WF, Alcon, Fort Worth, TX, USA) implantation for patients with early glaucoma (average mean deviation -1.6±2.4dB in the EDOF group and -2.4±2.0 in the monofocal group). They found that the Acrysof IQ Vivity provided significantly better intermediate and near vision, with similar distance vision and visual disturbance compared to the monofocal IOLs, and were associated with higher levels of spectacle independence and patient satisfaction in their cohort (19). Another study by Bissen-Miyajjma et al. compared EDOF IOLs and monofocal IOL outcomes in mild-moderate POAG eyes (average mean deviation of -10dB or better, without central visual field defects) with stable IOP on medical treatment, and found no significant difference between both groups for average mean deviation values, corrected distance visual acuity and contrast sensitivity (20).

One case series in Japan studied outcomes of diffractive EDOF IOLs (Symfony ZXROOV, Johnson & Johnson Surgical Vision, Santa Ana, CA, USA) in normal tension glaucoma (NTG) patients. The patients were well-controlled on a maximum of 2 glaucoma drops, had no central visual field defects and an average mean deviation of -4.78dB (range -0.79dB to -12.25dB) on Humphrey visual field testing. EDOF IOLs were implanted in 16 NTG eyes of 10 patients and had comparable outcomes with non-glaucomatous eyes with the same IOL implant for distance visual acuity and contrast sensitivity. However, the authors stressed that careful patient selection is required when considering EDOF implantation in NTG patients, to ensure that they have no central loss of visual field sensitivity, and that their NTG is stable and well-controlled on minimal treatment (21).

## MIGS and EDOF IOL implantation

The advent of EDOF IOLs comes in tandem with the boom in minimally-invasive glaucoma surgical (MIGS) devices. Trabecular-bypass MIGS devices such as the iStent inject (Glaukos Corporation, Aliso Viejo, CA, USA) and the Hydrus microstent (Alcon, Fort Worth, TX USA), used in glaucoma patients undergoing cataract surgery are astigmatically neutral, and have been shown to have predictable outcomes when combined with toric IOL implantation in glaucomatous eyes (22). In contrast, trabeculectomy surgery is associated with considerable with-the-rule astigmatic change in the immediate postoperative period which gradually shifts to against-the-rule astigmatism (6). Similarly, patients undergoing combined cataract and glaucoma drainage device surgery has also been shown to have more surgically-induced astigmatism and hyperopic surprise, due to the tendency for axial length to decrease after filtering surgery, compared to patients undergoing cataract surgery alone (23). Other bleb-forming MIGS, such as the Ex-Press glaucoma implant (Alcon, Fort Worth, TX, USA) and PreserFlo Microshunt (Santen Pharmaceutical Co., Osaka, Japan) have also been reported to induce cornea keratometric and topographical changes in the early post-operative period (24, 25). With Trabecular MIGS, glaucoma surgeons can consider a wider range of IOL options, including toric IOLs, during combined phacoemulsification and trabecular MIGS surgeries. A study by Ferguson et al. studied the outcomes of bilateral EDOF (AcrySof IQ Vivity or AcrySof IQ Vivity Toric, Alcon, Fort Woth, TX, USA) implantation in 52 eyes of 26 patients with mild (pre-perimetric) open-angle glaucoma and visually significant cataracts. 20 had concurrent trabecular microbypass (iStent inject, Glaukos Corporation, Aliso Viejo, CA, USA) implanted at the same time. Their subjects achieved favourable distance and intermediate vision (65% >= 20/20 unaided distance, 77% >= 29/32 unaided intermediate vision), with 92% not requiring glasses for driving and 50% not requiring glasses for computer work, and favourable mean Pelli-Robson contrast sensitivity of 1.78 +/- 0.17, which is comparable to a monofocal IOL (26).

## Important considerations for using EDOF IOLs in glaucoma patients

When treating glaucoma patients with cataract, important factors to consider include the extent of glaucoma damage, rate of glaucoma progression, patient’s lifestyle needs and their personal preferences. Caution should be exercised when treating advanced glaucoma patients with significantly constricted fields, NTG patients with central or paracentral visual field defects, patients with poorly-controlled intraocular pressures or rapidly progressing glaucoma. In such circumstances, the monofocal IOL would be the lens of choice, as it offers maximum image sharpness at a defined focal point and good contrast sensitivity, with the least visual disturbances. Additionally, EDOF IOLs are still considered ‘premium lenses’ and are more costly than the traditional monofocal IOLs. This is an important consideration for glaucoma patients, some of whom may require additional surgical procedures and possible life-long medical treatment in addition to cataract surgery.

EDOF IOLs are best-suited for stable glaucoma patients with mild disease who have preserved central visual fields, contrast sensitivity, and can also be used in conjunction with MIGS procedures. Consideration of a mini-monovision with bilateral EDOF implantation can also be considered in this group of patients, to help them achieve a higher rate of spectacle-independence for intermediate and near. It is also important to also be aware of the type of EDOF IOL technology used. For example, for the Acrysof IQ Vivity, the x-wave wavefront-shaping technology, which helps to stretch the wavefront to provide a continuous extended wave of vision, is incorporated into the central 2.2mm of the IOL (9, 27). Therefore, as pupil-size influences the patient’s post-operative vision outcome, studies have recommended performing dynamic pupillometry pre-operatively to minimise post-operative visual disturbance complaints (27).

The evolution of EDOF IOL technology is promising and has introduced more options for managing our glaucoma patients with cataracts. However, it remains important to individualise treatment decisions, particularly for glaucoma patients, who come with additional considerations related to the natural history of the disease, and to also manage patient expectations, as not all patients achieve spectacle-independence for intermediate and near distances, even with mini-monovision. Studies reporting the use of EDOF IOLs in cataract surgery for glaucoma patients are also limited, and more studies are required in this field, especially for different and newer types of EDOF IOLs that have been recently released.
